# Association between the Adherence to the International Guidelines for Cancer Prevention and Mammographic Density

**DOI:** 10.1371/journal.pone.0132684

**Published:** 2015-07-24

**Authors:** Adela Castelló, Leandro Prieto, María Ederra, Dolores Salas-Trejo, Carmen Vidal, Carmen Sánchez-Contador, Carmen Santamariña, Carmen Pedraz, Pilar Moreo, Nuria Aragonés, Beatriz Pérez-Gómez, Virginia Lope, Jesús Vioque, Marina Pollán, DDM-Spain research group

**Affiliations:** 1 Cancer Epidemiology Unit, National Center for Epidemiology, Instituto de Salud Carlos III, Madrid, Spain; 2 Consortium for Biomedical Research in Epidemiology & Public Health (CIBERESP), Instituto de Salud Carlos III, Madrid, Spain; 3 Cancer Epidemiology Research Group, Oncology and Hematology Area, IIS Puerta de Hierro (IDIPHIM), Madrid, Spain; 4 Navarre Breast Cancer Screening Program, Public Health Institute, Pamplona, Spain; 5 Valencian Breast Cancer Screening Program, General Directorate of Public Health, Valencia, Spain; 6 Cancer Prevention and Control Unit, Catalonian Institute of Oncology (ICO. Barcelona, Spain; 7 Balearic Islands Breast Cancer Screening Program, Regional Authority for Health & Consumer Affairs, Palma de Mallorca, Islas Baleares; 8 Galician Breast Cancer Screening Program, Galician Regional Health Authority, A Coruña, Spain; 9 Castile-León Breast Cancer Screening Program, General Directorate of Public Health, Burgos, Spain; 10 Aragón Breast Cancer Screening Program, Aragon Health Service, Zaragoza, Spain; 11 Universidad Miguel Hernandez, Sant Joan D'Alacant, Spain; Wayne State University School of Medicine, UNITED STATES

## Abstract

**Introduction:**

Mammographic density (MD) is considered a strong predictor of Breast Cancer (BC). The objective of the present study is to explore the association between MD and the compliance with the World Cancer Research Fund and the American Institute for Cancer Research (WCRF/AICR) recommendations for cancer prevention.

**Methods:**

Data of 3584 women attending screening from a population-based multicenter cross-sectional study (DDM-Spain) collected from October 7, 2007 through July 14, 2008, was used to calculate a score that measures the level of compliance with the WCRF/AICR recommendations: R1)Maintain adequate body weight; R2)Be physically active; 3R)Limit the intake of high density foods; R4)Eat mostly plant foods; R5)Limit the intake of animal foods; R6)Limit alcohol intake; R7)Limit salt and salt preserved food intake; R8)Meet nutritional needs through diet. The association between the score and MD (assessed by a single radiologist using a semi-quantitative scale) was evaluated using ordinal logistic models with random center-specific intercepts adjusted for the main determinants of MD. Stratified analyses by menopausal status and smoking status were also carried out.

**Results:**

A higher compliance with the WCRF/AICR recommendations was associated with lower MD (OR_1-unit increase_ = 0.93 95%CI:0.86;0.99). The association was stronger in postmenopausal women (OR = 0.91 95%CI:0.84;0.99) and nonsmokers (OR = 0.87;95%CI:0.80;0.96 for nonsmokers, OR = 1.01 95%CI:0.91;1.12 for smokers, *P*-interaction = 0.042). Among nonsmokers, maintaining adequate body weight (OR = 0.81 95%CI:0.65;1.01), practicing physical activity (OR = 0.68 95%CI:0.48;0.96) and moderating the intake of high-density foods (OR = 0.58 95%CI:0.40;0.86) and alcoholic beverages (OR = 0.76 95%CI:0.55;1.05) were the recommendations showing the strongest associations with MD.

**Conclusions:**

postmenopausal women and non-smokers with greater compliance with the WCRF/AICR guidelines have lower MD. These results may provide guidance to design specific recommendations for screening attendants with high MD and therefore at higher risk of developing BC.

## Introduction

The World Cancer Research Fund and the American Institute of Cancer Research (WCRF/AICR) estimate that around one fourth of cancer cases from high and medium income countries are preventable by adopting healthier lifestyles concerning diet, physical activity and body fatness. Regarding Breast Cancer (BC), published evidence indicates that between 20% and 42% of cases could be prevented in countries such as the USA, the UK, Brazil and China [1]. Taking into account that breast tumours are the most common cancer among women and one of the main causes of adult female mortality in developed countries [2], preventive strategies are of special importance. The WCRF/AICR issued in 2007, 8 general and 2 special recommendations on diet, physical activity and weight management for cancer prevention based on the available evidence [3, 4]. Five studies have already explored the association between compliance with such recommendations and BC risk, showing a beneficial effect [5–9].

A high mammographic density (MD), i.e. a high percentage of dense breast tissue, is considered a strong risk factor for BC [10, 11]. MD has also been associated with some of the WCRF/AICR recommendations such as body fatness [12, 13], diet[14, 15] and other obstetric and gynecological factors [16, 17], although, to our knowledge, no studies have investigated the relationship between adherence to the WCRF/AICR guidelines and MD. In this paper, the association between compliance with these recommendations and MD was explored in a group of Spanish women attending population-based BC screening programs, globally and separately according to their menopausal status. Finally, given the antiestrogenic effect of tobacco [18, 19], we hypothesized that this association might be different in smokers and nonsmokers. Therefore, we also explored the relationship between these recommendations and MD by smoking status.

## Methods

### Study population and data collection

The DDM-Spain study (*Determinantes de la Densidad Mamográfica en España-* Determinants of Mammographic Density in Spain) is a cross-sectional multicenter study including seven specific screening centers within the Spanish Breast Cancer Screening network located throughout the Spanish territory [13, 16]. All women aged 50–69 (45–69 in some regions), regardless of nationality or legal status, are screened under these government-sponsored programs every 2 years. Considering an initial prevalence of 25% of women with high MD, sample size was estimated as 3500 women (at least 500 women per center). This sample size allows identifying effects equal or greater than 1.25 for exposures over 40% with a statistical power of 80%. Women were randomly selected among screening attendants and invited to participate on a daily basis, until reaching the minimum sample size fixed for each center (500 women). A total of 3,584 women were recruited, with an average participation rate of 74.5% (range 64.7–84.0% across centers).Women were interviewed at the screening center by trained interviewers that collected demographic, anthropometric, physical activity, gynecologic, obstetric and occupational data, as well as family and personal history (including weight and height at age 18). Smoking information included current status and months since quitting for ex-smokers. Current smokers were defined as those women who smoke at the time of mammography or quit less than 6 months before. Dietary intake during the preceding year was collected using a validated 95-items food frequency questionnaire (FFQ) [20, 21]. Post-menopausal status was defined as self-reported absence of menstruation in the last 12 months. Interviewers measured weight, height, waist and hip circumferences twice using the same protocol and identical balance scales, stadiometers and measuring tapes. A third measure was taken when the first two were not similar. MD was assessed by a single radiologist from the craniocaudal mammogram of the left breast using a visual semiquantitative score with six categories proposed by Boyd [22], namely, A (0%), B (0–10%), C (10–25%), D (25–50%), E (50–75%) and F (>75%). Given the small percentage of women in category A (4%) the two lowest categories were grouped together, creating the definitive outcome variable categorized as: <10%, 10–25%, 25–50%, 50–75% and >75%.

After excluding 10 women who developed breast cancer within 6 months of mammography, 16 women without MD assessment and 8 women with a daily kcal intake under 750 or above 4500, information on 3,550 women was considered in the analyses. In the remaining sample, the mean calorie intake was 2054 (Min-Max: 835–4246).

### Ethics statement

The DDM-Spain study protocol was formally approved by the bioethics and animal welfare committee at the Carlos III Institute of Health and all participants signed a consent form, including permission to publish the results from the current research.

### WCRF/AICR score

Based on the WCRF/AICR guidelines [3, 4] and following the methodology described in previous studies [5–9]. A score was constructed considering the 8 general recommendations (**Table 1**): R1) body fatness, R2) physical activity, R3) foods and drinks that promote weight gain, R4) plant foods, R5) animal foods, R6) alcoholic drinks, R7) preservation, processing and preparation of foods and R8) dietary supplements. The breast is a fat storage area, therefore MD heavily depends on body mass index (BMI)[23]. For this reason, BMI at the time of mammography was considered as a confounder and was not included as part of the score. However, the association between MD and body fatness during adolescence is still not clear [24] and several studies report a positive association between adult weight gain and MD [13, 25, 26]. Accordingly, we decided to include BMI at age 18 and weight gained during adulthood to calculate the subscore for R1. Special recommendation S1 was not included because breastfeeding seem to be positively associated with MD [16, 17], therefore the protective effect of breastfeeding on BC is not likely to act through its association with MD. Finally, special recommendation for cancer survivors S2), was not applicable to this population.

**Table 1:** Operationalization of the WCRF/AICR recommendations for cancer prevention in a score (0–8) using DDM-Spain study data.

WCRF/AICR RECOMMENDATIONS	PERSONAL RECOMMENDATIONS	OPERATIONALIZATION^a^	SCORE
1) Body fatness. Be as lean as possible without becoming underweight	**BMI(in kg/m2) 18years?**	18.5–25 Kg/m2	1
	1a) Ensure that body weight through childhood and adolescence growth projects towards the lower end of normal BMI range at age 18y	25–30 Kg/m2	0.5
		<18.5 or ≥30 Kg/m2	0
	**Weight gain per 10 years from 18years?**	≤2.5 Kg/10years	1
	1b) Maintain body weight within the normal range from age 18y	2.5–5 Kg/10years	0.5
	1c) Avoid weight gain and increases in waist circumference throughout adulthood	>5Kg/10years	0
2) Physical activity. Be physically active as part of your everyday life	**Walking/cycling (min/day)**	>40 min/d	1
	2a) Be moderately physically active, equivalent to brisk walking, for > = 30min every day	20–40 min/d	0.5
		<20 min/d	0
	**Exercise/Sports (h/week)**	≥4h/week	1
	2b) As fitness improves, aim for > = 60 min of moderate or for > = 30 min of vigorous physical activity every day	2–4h/week	0.5
		<2h/week	0
	**Watching TV/using computer/reading (h/day)**	≤2h/d	1
	2c) Limit sedentary habits such as watching television	2–4h/d	0.5
	** **	≥4h/d	0
3) Foods and drinks that promote weight gain. Limit consumption of energy-dense foods; avoid sugary drinks	**Energy-dense foods (Kcal/g)** ^**b**^	<125kcal/100 g	1
	3a) Consume energy-dense foods sparingly	125-175kcal/100 g	0.5
		≥175Kcal/100 g	0
	**Sugary drinks intake (g/day)** ^**c**^	0 g/d	1
	3b) Avoid sugary drinks	0–250 g/d	0.5
		>250 g/d	0
	**Fast food intake (g/day)** ^**d**^	<7 g/d (p33)	1
	3c) Consume fast foods sparingly	7–15 g/d	0.5
		≥15 g/d (p66)	0
4) Plant foods. Eat mostly foods of plant origin	**Fruits and Vegetables (g/day)** ^**e**^	≥400 g/d	1
	4a) eat> = 5 portions/servings(> = 400 g) of a variety of non-starchy vegetables and fruit every day	200–400 g/d	0.5
		<200 g/d	0
	**Cereals, whole grain bread and legumes (g/day)** ^**f**^	≥ 60 g/d (p66)	1
	4b) Eat relatively unprocessed cereals (grains) and/or pulses(legumes) with every meal	20–60 g/d	0.5
		<20 g/d (p33)	0
	**White bread, pasta and rice (g/day)** ^**g**^	<83 g/d (p33)	1
	4c) Limit refined starchy food	83–168 g/d	0.5
		≥168 g/d (p66)	0
	4d) people who consume starchy roots or tubers as staples should also ensure sufficient intake of no starchy vegetables, fruit and pulses (legumes)	Not sufficient data available	
5) Animal foods. Limit intake of red meat and avoid processed meat	**Red (R) and processed (P) meat** ^**h**^	R+P<500g/wk and P<3g/d	1
	People who eat red meat should consume <500g/wk and very few, if any, processed meat	R+P<500g/wk and P 3–50g/d	0.5
		R+P≥500g/wk or P≥50 g/d	0
6) Alcoholic drinks. Limit alcoholic drinks	**Ethanol intake (g/day)** ^**i**^	≤10 g/d	1
	If alcoholic drinks are consumed, limit consumption to < = 1 drink/d	10–20 g/d	0.5
		≥20 g/d	0
7) Preservation, processing, preparation. Limit consumption of salt. Avoid moldy cereals (grains or pulses)	**Cold Meat & Salted/Smoked fish** ^**j**^	<20g/d (p33)	1
	7 a) Avoid slat-preserved, salted or salty foods. Preserve foods without using salt	21–30 g/d	0.5
		>30 g/d (p66)	0
	**Sodium**	<2.4 g/d	1
	7b) Limit consumption of processed foods with added salt to ensure an intake of sodium <2.4 g/d	2.4–3 g/d	0.5
		≥3 g/d	0
	7c) Do not eat moldy cereals (grains) or pulses (legumes)	Insufficient data available	
8) Dietary supplements. Aim to meet nutritional needs through diet alone WCRF/AICR especial recommendations	**Supplement use**	No	1
	Dietary supplements are not recommended for cancer prevention	1 /day	0.5
		>1/day	0
S1) Breastfeeding. Mothers to breastfeed; children need to be breastfeed	**Cumulative breastfeeding**	** **	
	Aim to breastfeed infants exclusively up to 6 months and continue with complementary feeding thereafter	Not applicable to this population	
		
S2) Cancer survivors. Follow the recommendations for cancer prevention		Not applicable to this population	

^a^ Cutoffs provided in the “World Cancer Research Fund / American Institute for Cancer Research. Food, Nutrition, Physical Activity, and the Prevention of Cancer: a Global Perspective. Washington DC: AICR, 2007. In. 2007; 289–295.” or in the distribution of the data when the cut point was not specified

^b^ Energy intake for all foods considered

^c^ Sugary drinks: juices and other sugared beverages.

^d^ Fast food: Fried potatoes, crisps, pizza, chicken and Serrano ham croquette, mayonnaise and ketchup

^e^ Fruits and vegetables: Spinach, chard, lettuce, endive, escarole, tomato, onion, carrot, carrot, pumpkin, cooked cabbage, green beans, eggplant, zucchini, cucumber, pepper, artichoke, asparagus, mushroom, garlic, orange, banana, apple, pear, water melon, grapes, plums or prunes (dried or fresh), strawberry and kiwi.

^f^ Cereals: whole grain bread and cereals.

^g^ Refined Grains: White bread, pasta and rice.

^h^ Red and processed meat: Pork, beef, lamb, liver (beef, pork or chicken), entrails, hamburger, serrano ham and other cold meat, sausage, bacon, pâte, foie-gras.

^i^ Alcohol: Measured as total ethanol intake coming from wine, beer and spirits.

^j^ Serrano ham and other cold meat and smoked and salt preserved fish.

For each item considered under each recommendation a maximum score of 1 was assigned when the item was fully met, an intermediate score of 0.5 when the item was not far from being met and 0 points otherwise. This decision was taken based on the cutoffs provided in WCRF/AICR report [3] or on the distribution of the data when the cutoff was not specified. The score for recommendations including several items was calculated as the average of their marks. Each recommendation was considered to contribute equally to the final index that was calculated as the sum of the individual scores. Therefore, the WCRF/AICR score ranged 0–8 and represented the minimum number of recommendations fully met. This score was grouped into 4 categories as follows: 0 to <4, 4 to <5, 5 to <6, 6 to 8. The cut points were selected ensuring a sufficient number of women in each category and following the methodology proposed by previous research [5–9].

### Statistical methods

The association between MD and the WCRF/AICR score was evaluated using ordinal logistic models with random center-specific intercepts. These models were adjusted for a set of potential confounders including age, BMI, parity, family history of breast cancer, use of hormonal replacement therapy (HRT), menopausal status and smoking habit. The categorical and continuous associations with the index were evaluated. Separate analyses were performed according to menopausal status (pre- and post-menopausal) and tobacco consumption (smokers and nonsmokers). Heterogeneity of effects was tested including in the model an interaction term between the score and menopausal or smoking status. In all instances, non-linear associations were explored using natural splines, with 5 knots located in Harrell’s recommended percentiles, namely, 5, 27.5, 50, 72.5 and 95% confidence intervals (CI) [27].

Same analyses were carried out to evaluate the association between MD and each specific recommendation. These models were adjusted for the set of variables described above plus the effect of the rest of the recommendations. To do so, the sum of the scores for all recommendations excluding the one under study was calculated and used as a potential confounder.

All analyses were performed using Stata statistical software (version MP 12.1; Stata Corp LP, College Station, TX). The last statistical analyses were conducted in August 2014.

## Results

**Table 2** shows the distribution of the overall WCRF/AIRC score and individual recommendations as well as some baseline characteristics of the study sample stratifying by menopausal and smoking status. Postmenopausal women showed a higher adherence to the WCRF/AIRC guidelines, particularly to those related to body fatness, consumption of high density, plant, animal and salty foods. Nonsmokers also seemed to have better lifestyle habits than smokers concerning the intake of high density, plant and animal foods, as well as alcohol consumption. Regarding baseline characteristics, as expected, postmenopausal women showed lower MD, higher age, BMI, number of deliveries and use of HRT than their premenopausal counterparts. MD was lower among nonsmokers who were also older, with higher BMI and had greater number of deliveries than smokers. For interested readers, S1 Table summarizes the distribution (mean and standard error) of the WCRF/AIRC score and of each specific recommendation per categories of MD, adjusted by age, BMI and center.

**Table 2:** Description of all women’s characteristics and by menopausal status and by smoking habit.

	ALL WOMEN	PREMENOPAUSAL	POSTMENOPAUSAL		NONSMOKER OR FORMER +6MO	SMOKER OR FORMER <6MO	
	n = 3550	n = 816	n = 2734	p	n = 2180	n = 1370	p
**Score**				<0.001			0.41
**0 to <4**	477(13%)	126(15%)	351(13%)		279(13%)	198(14%)	
**4 to <5**	1287(36%)	332(41%)	955(35%)		803(37%)	484(35%)	
**5 to <6**	1286(36%)	271(33%)	1015(37%)		798(37%)	488(36%)	
**6 to 8**	500(14%)	87(11%)	413(15%)		300(14%)	200(15%)	
**R1)Maintain adequate body weight**				0.003			<0.001
**Not Met** ^**a**^	339(10%)	88(11%)	251(9%)		232(11%)	107(8%)	
**Not far from Met** ^**a**^	1106(31%)	284(35%)	822(30%)		673(31%)	433(32%)	
**Met** ^**a**^	1555(44%)	344(42%)	1211(44%)		885(41%)	670(49%)	
Missing*	550(15%)	100(12%)	450(16%)		390(18%)	160(12%)	
**R2)Be physically active**				0.145			0.241
**Not Met** ^**a**^	287(8%)	53(7%)	234(9%)		169(8%)	118(9%)	
**Not far from Met** ^**a**^	2621(74%)	608(75%)	2013(74%)		1631(75%)	990(72%)	
**Met** ^**a**^	642(18%)	155(19%)	487(18%)		380(17%)	262(19%)	
**R3)Limit intake of high density foods**				<0.001			0.001
**Not Met** ^**a**^	57(1.61%)	18(2%)	39(1%)		27(1%)	30(2%)	
**Not far from Met** ^**a**^	1904(54%)	512(63%)	1392(51%)		1131(52%)	773(56%)	
**Met** ^**a**^	1589(45%)	286(35%)	1303(48%)		1022(47%)	567(41%)	
**R4) Eat mostly plant foods**				0.014			0.017
**Not Met** ^**a**^	108(3%)	21(3%)	87(3%)		55(3%)	53(4%)	
**Not far from Met** ^**a**^	2159(61%)	532(65%)	1627(60%)		1309(60%)	850(62%)	
**Met** ^**a**^	1283(36%)	263(32%)	1020(37%)		816(37%)	467(34%)	
**R5)Limit intake of animal foods**				<0.001			0.022
**Not Met** ^**a**^	1412(40%)	406(50%)	1006(37%)		841(39%)	571(42%)	
**Not far from Met** ^**a**^	2069(58%)	399(49%)	1670(61%)		1304(60%)	765(56%)	
**Met** ^**a**^	69(2%)	11(1%)	58(2%)		35(2%)	34(2%)	
**R6)Limit alcohol intake**				0.143			<0.001
**Not Met** ^**a**^	170(5%)	38(5%)	132(5%)		74(3%)	96(7%)	
**Not far from Met** ^**a**^	444(13%)	86(11%)	358(13%)		265(12%)	179(13%)	
**Met** ^**a**^	2936(83%)	692(85%)	2244(82%)		1841(84%)	1095(80%)	
**R7)Limit salt intake and salt preserved food consumption**				<0.001			0.069
**Not Met** ^**a**^	903(25%)	271(33%)	632(23%)		528(24%)	375(27%)	
**Not far from Met** ^**a**^	1625(46%)	364(45%)	1261(46%)		1026(47%)	599(44%)	
**Met** ^**a**^	1022(29%)	181(22%)	841(31%)		626(29%)	396(29%)	
**R8)Meet nutritional needs through diet alone**				0.059			0.072
**Not Met** ^**a**^	87(2%)	13(2%)	74(3%)		55(3%)	32(2%)	
**Not far from Met** ^**a**^	384(11%)	77(9%)	307(11%)		256(12%)	128(9%)	
**Met** ^**a**^	3079(87%)	726(90%)	2353(86%)		1869(86%)	1210(88%)	
**% of Dense Tissue (Boyd)**				<0.001			<0.001
**<10%**	871(25%)	107(13%)	764(28%)		600(28%)	271(20%)	
**10–25%**	733(21%)	108(13%)	625(23%)		460(21%)	273(20%)	
**25–50%**	1136(32%)	268(33%)	868(32%)		701(32%)	435(32%)	
**50–75%**	623(18%)	252(31%)	371(14%)		325(15%)	298(22%)	
**>75%**	187(5%)	81(10%)	106(4%)		94(4%)	93(7%)	
**Age mean(SD)**	56.20(5.46)	49.83(2.87)	58.10(4.53)	<0.001	57.68(5.12)	53.85(5.14)	<0.001
**BMI mean(SD)**	28.03(4.99)	27.00(4.94)	28.34(4.97)	<0.001	28.69(4.86)	26.98(5.02)	<0.001
**Number of deliveries mean(SD)**				<0.001			<0.001
**0**	316(9%)	83(10%)	233(9%)		155(7%)	161(12%)	
**1**	524(15%)	169(21%)	355(13%)		265(12%)	259(19%)	
**2**	1686(47%)	411(50%)	1275(47%)		1050(48%)	636(46%)	
**> = 3**	1024(29%)	153(19%)	871(32%)		710(33%)	314(23%)	
**Family history of BC n(%)**				0.935			0.503
**No**	3291(93%)	757(93%)	2534(93%)		2026(93%)	1265(92%)	
**Yes**	259(7%)	59(7%)	200(7%)		154(7%)	105(8%)	
**Use of HRT n(%)**				<0.001			0.972
**No**	3202(90%)	808(99%)	2394(88%)		1966(90%)	1236(90%)	
**Yes**	348(10%)	8(1%)	340(12%)		214(10%)	134(10%)	

^**a**^Defined as: Not met = score in recommendation <0.25; Not far from met = score in recommendation 0.25–075; Met = score in the recommendation ≥0.75.

The relationship between adherence to the WCRF/AICR guidelines and MD for all women and stratifying by menopausal and smoking status is summarized in **Table 3**. Overall, a clear inverse association between the score and MD was observed and the linear trend was found to be statistically significant (Odds ratio (OR) = 0.93; 95%CI = 0.86;0.99; p-trend = 0.019). The interaction between the score and menopausal status was not significant (p-int = 0.446), but a clear association was only observed in postmenopausal women (OR = 0.91; 95%CI = 0.84;0.99; p-trend = 0.017). On the other hand, the relationship between MD and the score was significantly different among smokers and non-smokers (p-int = 0.042). Thus, while the relationship between the WCRF/AICR score and MD was statistically significant in non-smokers (OR = 0.87; 95%CI = 0.80; 0.96; p- trend = 0.002), no association was observed in smokers (OR = 1.01; 95%CI = 0.91; 1.12; p- trend = 0.965).

**Table 3:** Association between the WCRF/AICR score and MD in all women, by menopausal status and by smoking habit.

	ALL WOMEN		PREMENOPAUSAL		POSTMENOPAUSAL			NONSMOKER OR FORMER +6MO		SMOKER OR FORMER <6MO		
	n = 3550		n = 816		n = 2734			n = 2180		n = 1370		
	n	OR^a^(95%CI)	n	OR^b^(95%CI)	n	OR^b^(95%CI)	p-int	n	OR^c^(95%CI)	n	OR^c^(95%CI)	p-int
0 to <4	477	1	126	1	351	1		279	1	198	1	
4 to <5	1286	0.96 (0.79;1.17)	332	0.90 (0.62;1.30)	954	0.99 (0.79;1.24)		803	0.83 (0.65;1.07)	483	1.16 (0.86;1.56)	
5 to <6	1285	0.87 (0.72;1.06)	271	0.85 (0.58;1.25)	1014	0.88 (0.70;1.10)		797	0.74 (0.58;0.96)	488	1.07 (0.80;1.44)	
6 to 8	500	0.79 (0.63;1.00)	87	0.91 (0.56;1.50)	413	0.77 (0.59;1.01)		300	0.64 (0.47;0.87)	200	1.05 (0.73;1.49)	
p-trend		0.019		0.583		0.017			0.002		0.965	
1-unit increase		0.93 (0.86;0.99)		0.97 (0.84;1.13)		0.91 (0.84;0.99)	0.446		0.87 (0.80;0.96)		1.01 (0.91;1.12)	0.042

^a^ Adjusted by age, BMI, number of deliveries, family history of BC, use of HRT, menopausal status and smoking habit

^b^ Adjusted by age, BMI, number of deliveries, family history of BC, use of HRT and smoking habit

c Adjusted by age, BMI, number of deliveries, family history of BC, use of HRT and menopausal status.

**Fig 1:**
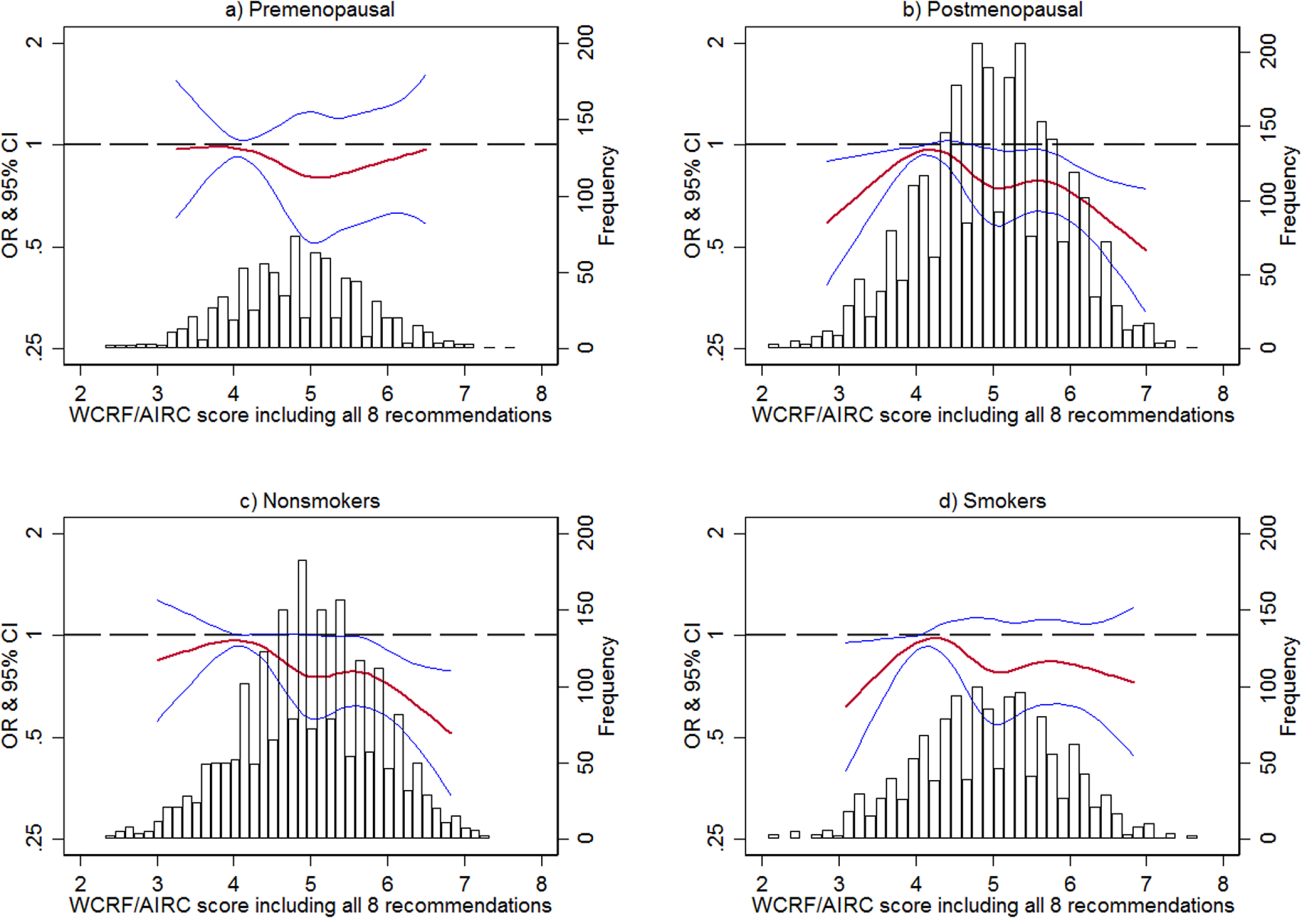
Dose-response shape (natural splines) of the association between the WCRF/AICR score and MD stratifying by menopausal status and smoking habit. The first quintile of the score has been taken as reference.

Analysis of the dose-response shape is presented in **Fig 1**. A clear trend was only seen in the postmenopausal group and in those who were nonsmokers (overall trend p-values of 0.004 and 0.023 respectively). Regarding postmenopausal women, there was a statistically significant departure from linearity (p-value = 0.013), meaning that, a downward trend of MD with the WCRF/AICR score was only seen in women who met at least 5 recommendations. On the other hand, the dose-response curve was linear for non-smokers.

The analysis of the association between individual recommendations and MD (**Table 4**) revealed that an adequate body fatness throughout life (OR = 0.84; 95%CI = 0.70;1.00) and a moderate consumption of high density foods (OR = 0.75; 95%CI = 0.56;1.01) seem to be marginally associated with a reduced MD, especially in postmenopausal women (OR = 0.84; 95%CI = 0.69;1.03 and OR = 0.71; 95%CI = 0.51;0.99 respectively) for which avoiding excessive alcohol consumption also appeared to be an important factor (OR = 0.80; 95%CI = 0.61;1.04). Among nonsmokers, a reduced MD was observed in women with an adequate body fatness throughout life (OR = 0.81; 95%CI = 0.65;1.01),those with moderate consumption of high density foods (OR = 0.58; 95%CI = 0.40;0.86) and those avoiding excessive alcohol consumption (OR = 0.76; 95%CI = 0.55;1.05). Additionally, physical activity was also inversely associated with MD in this subgroup (OR = 0.68; 95%CI = 0.48;0.96). Interestingly, the interaction term with smoking status was significant for physical activity and for intake of high density foods, being the inverse association only observed among the nonsmokers’ group.

**Table 4:** Association between individual recommendations and MD in all women, by menopausal status and by smoking habit.

	ALL WOMEN	PREMENOPAUSAL	POSTMENOPAUSAL		NONSMOKER OR FORMER	SMOKER OR FORMER	
	n = 3550	n = 816	n = 2734		+6MO n = 2180	<6MO n = 1370	
RECOMMENDATIONS	OR^a^(95%CI)	OR^b^(95%CI)	OR^b^(95%CI)	p-int	OR^c^(95%CI)	OR^c^(95%CI)	p-int
R1) **Maintain adequate body weight**	0.84 (0.70;1.00)	0.82 (0.57;1.17)	0.84 (0.69;1.03)	0.888	0.81 (0.65;1.01)	0.89 (0.67;1.18)	0.606
R2) **Be physically active**	0.87 (0.66;1.14)	1.13 (0.65;1.98)	0.80 (0.59;1.09)	0.285	0.68 (0.48;0.96)	1.25 (0.83;1.89)	0.025
R3) **Limit intake of high density foods**	0.75 (0.56;1.01)	0.91 (0.50;1.66)	0.71 (0.51;0.99)	0.466	0.58 (0.40;0.86)	1.05 (0.67;1.63)	0.044
R4) **Eat mostly plant foods**	0.99 (0.76;1.28)	1.08 (0.61;1.90)	0.97 (0.73;1.29)	0.733	0.88 (0.64;1.22)	1.18 (0.78;1.76)	0.277
R5) **Limit intake of animal foods**	1.08 (0.85;1.38)	1.18 (0.73;1.92)	1.06 (0.80;1.39)	0.684	0.97 (0.72;1.32)	1.28 (0.88;1.86)	0.246
R6) **Limit alcohol intake**	0.85 (0.67;1.07)	1.05 (0.64;1.71)	0.80 (0.61;1.04)	0.327	0.76 (0.55;1.05)	0.95 (0.68;1.32)	0.342
R7) **Limit salt intake and salt preserved food consumption**	1.03 (0.85;1.24)	0.89 (0.60;1.31)	1.07 (0.87;1.33)	0.403	1.09 (0.86;1.39)	0.94 (0.70;1.25)	0.413
R8) **Meet nutritional needs through diet alone**	1.12 (0.84;1.50)	1.28 (0.65;2.53)	1.09 (0.79;1.50)	0.680	0.97 (0.68;1.39)	1.45 (0.90;2.35)	0.183

^a^ Adjusted by age, BMI, number of deliveries, family history of BC, use of HRT, menopausal status, smoking habit and compliance with the other recommendations

^b^ Adjusted by age, BMI, number of deliveries, family history of BC, use of HRT, smoking habit and compliance with the other recommendations

^c^ Adjusted by age, BMI, number of deliveries, family history of BC, use of HRT, menopausal status and compliance with the other recommendations.

## Discussion

Our study suggests that a higher compliance with the WCRF/AICR recommendations is associated with a lower MD in postmenopausal women. Regarding smoking, no associations were observed among women who smoked. Among nonsmokers, these recommendations and specifically those focused on maintaining adequate body fatness throughout life, practicing physical activity, avoiding consumption of high density foods and limiting alcohol consumption were associated with decreased MD.

To our knowledge, this is the first study exploring the association between MD and the adherence to the WCRF/AICR guidelines. However, our results are in agreement with the few studies that have assessed the relationship between these recommendations and BC risk [5–9]. All of them report a significant downward trend in BC risk as the number of recommendations met increases. Two of these studies found the strongest associations with the recommendations related to body fatness and alcohol intake [5, 8] and another two with energy dense foods [8, 9], findings that are in agreement with ours. Concerning the association between individual recommendations and MD, while current BMI is negatively correlated with MD, adult weight gain (Rec 1) seems to be positively associated as we have previously reported [13]. Two other studies reported similar results [25, 26], whereas a third suggested otherwise [28]. This inconsistency may be explained, at least in part, by the use of different tools to assess MD [13]. Regarding physical activity (Rec2) a recent review fails to identify a clear effect on breast density [29], something confirmed by recent works [30, 31]. In our study, a clear heterogeneous effect among smokers and nonsmokers was found that must be corroborated by others. Similarly, we found a negative association between lower consumption of energy-dense foods and sugary drinks and MD (Rec 3) among nonsmokers, that agrees with other published research [32–35]. Some studies suggest a possible inverse association between plant foods (Rec 4) and MD, mostly regarding vegetable consumption [7, 35–37], while others found no effect or a positive association between fruit intake and MD [7, 38]. The combination of these two items in a single category might explain the lack of any effect. In fact, in our study, the relationship between MD and vegetables and fruits and MD seem to go in different directions (being negative for vegetables and positive for fruits), but neither of them reached statistical significance (data not shown). The influence of red/processed meat consumption (Rec 5) on MD is not clear with the few existing studies suggesting either a positive [14, 35] or a null [33] relationship. As seen in our results, avoiding excessive intake of alcohol (Rec 6) might be associated with lower MD [34, 36], especially among postmenopausal women [34]. Regarding salty foods (Rec7), to our knowledge no previous studies have explored their association with MD, but breast cancer is not among the tumors that seem to benefit from this recommendation [3, 4]. Finally, among the three previous studies that explored the effect of dietary supplements on MD (Rec8), one of them reported an inverse relationship [37], while the other two suggested otherwise [34, 39]. Concerning the analysis by menopausal status, it is important to highlight that, even though the number of premenopausal women was insufficient to detect statistically significant differences between pre and postmenopausal screening attendants, a clear dose-response effect of the WCRF/AICR score was only seen in the last group. It is widely known that obesity increases the risk of BC only in menopausal women [3, 4]. It is possible that, as it is the case of body fatness and alcohol, these recommendations exert their effect influencing the levels of circulating estrogens and other hormones, such as insulin and insulin-like growth factor 1 [3, 4, 40]. Their impact on MD would be particularly important after menopause, when these variations of the hormonal milieu may influence the natural process of mammary involution [41].

Unfortunately, none of the above mentioned studies on WCRF/AICR recommendations and BC stratified by smoking status. In our study, the negative association of MD with the adherence to these guidelines was only seen in women who did not smoke. In this sense, we have previously described a relationship between alcohol consumption and MD that was only observed among nonsmokers [42] and other authors have found an interaction between the effect of smoking and obesity on breast cancer risk [43]. Estrogen level is an important mediator of the mechanism by which some of these risk factors exert their action [3, 4, 40]. The antiestrogenic effect of tobacco [18, 19] may explain the lack of association between the score and MD among smokers. On the other hand, cigarette smoke is known to contain over 7,000 chemicals, 69 of which are established carcinogens [44], including over 20 that are established mammary carcinogens [45]. Therefore, it is possible that the harmful effect of smoking counteracts the potential benefits of these recommendations.

DDM-Spain is the biggest study published up to date on risk factors and MD that contains complete dietary information and is the first exploring the effect of WCRF/AICR recommendations on MD. Nevertheless, our study also has some limitations. As mentioned before, in spite of the differences seen in the pre and postmenopausal subgroups, the number of premenopausal women was insufficient to reach conclusions for this particular subgroup. This limitation results from the age-groups targeted by Spanish screening programs that, with few exceptions, initiate screening at age 50 [46]. Secondly, even though screening participation rates are high [46], it is well known that screening attendants tend to be more concerned about their health than non-attendants, which may imply an underrepresentation of less compliant women in our study. However, our study included women from all socioeconomic levels, and the prevalence of different lifestyle factors, such as smoking, physical activity and use of hormonal treatment was very similar to that reported by the Spanish National Health Survey for women in the same age range [47]. Regarding data collection, the use of different mammographic devices and interviewers in different centers might introduce some heterogeneity. Random center-specific intercepts were used in order to account for these unmeasured sources of variability. Breast density was visually assessed by a single radiologist using a semi-quantitative scale on analog and digital mammograms, which may imply a degree of subjectivity. However, the evaluation of the intra-agreement of MD measurements was excellent [48], and we have confirmed that this visual scale is a risk predictor of subsequent BC development [11]. Finally, some methodological issues should be taken into account when interpreting the results. On the one hand, the cross-sectional design of the current study precludes the establishment of causal relationships between the adherence to the WCRF/AICR guidelines and MD. On the other hand, multiple testing is a concern in situations where a great number of tests are carried out. While this is not a problem in the analysis of our main objective, namely the association between global compliance with the WCRF/AICR guidelines and MD, it can be an issue in secondary analyses of individual recommendations. However, taking into account that we adjusted 24 models, an alpha error of 5% implies that chance would explain only 1 of the statistically significant results presented here.

## Conclusion

A high compliance with the WCRF/AICR guidelines, particularly maintaining an adequate weight throughout adult life, practicing physical activity, and limiting the consumption of alcohol, high density foods, and sugary drinks, is associated with a lower MD. More studies are needed to investigate the potential of these recommendations to reduce MD, one of the strongest risk factors for BC.

## Supporting Information

S1 Table Distribution of the total and individual scores for the WCRF/AICR recommendations for cancer prevention in women participating in DDM-Spain study by Boyd categories of mammographic density adjusted by age, bmi and center. (DOCX)
