# Supplementary material for: Association between the Adherence to the International Guidelines for Cancer Prevention and Mammographic Density
Source: PLoS One. 2015 Jul 24;10(7):e0132684. doi: 10.1371/journal.pone.0132684 (PMC4514863; doi:10.1371/journal.pone.0132684)
Supplement: S1 Table — (DOCX) [file pone.0132684.s001.docx]

**S1 Table.** Distribution of the total and individual scores for the WCRF/AICR recommendations for cancer prevention in women participating in DDM-Spain study by Boyd categories of mammographic density adjusted by age, bmi and center

|  |  |  | **BOYD** |  |  |  |
| --- | --- | --- | --- | --- | --- | --- |
|  | **<10%** | **10-25%** | **25-50%** | **50-75%** | **>75%** | **p** |
|  | n=871 | n=733 | n=1136 | n=623 | n=187 |  |
| **WCRF/AIRC Score Mean (EE)** | 5.01 (0.03) | 5.00(0.04) | 4.93(0.03) | 4.91(0.04) | 4.98(0.06) | 0.127 |
| **R1) Maintain adequate body weight Mean (EE)** | 0.53 (0.01) | 0.53(0.01) | 0.53 (0.01) | 0.50 (0.01) | 0.49(0.03) | 0.270 |
| **R2) Be physically active Mean (EE)** | 0.57 (0.01) | 0.56(0.01) | 0.55 (0.01) | 0.56 (0.01) | 0.55(0.02) | 0.236 |
| **R3) Limit intake of high density foods Mean (EE)** | 0.72 (0.01) | 0.70(0.01) | 0.69 (0.01) | 0.69 (0.01) | 0.73(0.02) | 0.010 |
| **R4) Eat mostly plant foods Mean (EE)** | 0.66 (0.01) | 0.63(0.01) | 0.65 (0.01) | 0.64 (0.01) | 0.68(0.02) | 0.110 |
| **R5) Limit intake of animal foods Mean (EE)** | 0.31 (0.01) | 0.32(0.01) | 0.31 (0.01) | 0.31 (0.01) | 0.32(0.02) | 0.678 |
| **R6) Limit alcohol intake Mean (EE)** | 0.91 (0.01) | 0.89(0.01) | 0.89 (0.01) | 0.88 (0.01) | 0.89(0.02) | 0.336 |
| **R7) Limit salt intake and salt preserved food consumption Mean (EE)** | 0.41 (0.01) | 0.43(0.01) | 0.40 (0.01) | 0.41 (0.01) | 0.42(0.02) | 0.592 |
| **R8) Meet nutritional needs through diet alone Mean (EE)** | 0.92 (0.01) | 0.93(0.01) | 0.92 (0.01) | 0.93 (0.01) | 0.91(0.02) | 0.399 |
